# Tumor microenvironment characterization in esophageal cancer identifies prognostic relevant immune cell subtypes and gene signatures

**DOI:** 10.18632/aging.203800

**Published:** 2021-12-26

**Authors:** Yuhong Zhang, Minqi Zhu, Junxian Mo, Lei Xian

**Affiliations:** 1 Department of Gastroenterology, The First Affiliated Hospital of Guangxi Medical University, Nanning, China; 2 Guangxi Medical University, Nanning, China; 3 Department of Cardiothoracic Surgery, The Second Affiliated Hospital of Guangxi Medical University, Nanning, China

**Keywords:** esophageal cancer, tumor microenvironment, prognostic model, immune cell subtypes, bioinformatics

## Abstract

Esophageal cancer (ESCA) is a common malignancy in the digestive system with a high mortality rate and poor prognosis. Tumor microenvironment (TME) plays an important role in the tumorigenesis, progression and therapy resistance of ESCA, whereas its role in predicting clinical outcomes has not been fully elucidated. In this study, we comprehensively estimated the TME infiltration patterns of 164 ESCA patients using Gene Set Variation Analysis (GSVA) and identified 4 key immune cells (natural killer T cell, immature B cell, natural killer cell, and type 1 T helper cell) associated with the prognosis of ESCA patients. Besides, two TME groups were defined based on the TME patterns with different clinical outcomes. According to the expression gene set between two TME groups, we built a model to calculate TMEscore based on the single-sample gene-set enrichment analysis (ssGSEA) algorithm. TMEscore systematically correlated the TME groups with genomic characteristics and clinicopathologic features. In conclusion, our data provide a novel TMEscore which can be regarded as a reliable index for predicting the clinical outcomes of ESCA.

## INTRODUCTION

Esophageal cancer (ESCA) is a common malignancy in the digestive system with an extremely aggressive nature and poor prognosis [1]. It is estimated that ESCA is the 8th most common cancer and the 6th leading cause of cancer-specific death [2, 3]. Although esophagectomy is still considered the cornerstone of curative treatment for locally advanced esophageal cancer, it remains associated with considerable postoperative morbidity, mortality, and recurrence rates [4]. And the eligibility of a patient for surgical resection strongly depends on the extent of the disease, as well as on the general condition of the patient [5]. Nowadays, despite recent improvements in early diagnosis and therapeutic strategy, the overall survival (OS) of esophageal cancer patients remains lower than most solid tumors [6, 7].

As the novel therapeutic options, immune checkpoint inhibitors have been applied and revealed encouraging efficacy in ESCA [8]. Additionally, other immunogenic approaches such as adoptive T-cell therapy and peptide vaccines also exhibited promising curative effects [9, 10]. Due to the ideal results of targeting the immune microenvironment in the treatment of esophageal cancer, tumor microenvironment (TME) may play a crucial role in the tumorigenesis and progression of ESCA.

TME cells often take part in the occurrence and development of esophageal cancer.

The TME includes a complex collection of components, such as stromal cells with immunosuppressive features and several anti-tumor components, for example cytotoxic T lymphocytes, T helper type1 cells, and natural killer cells [11]. And some cells display both pro- and anti-tumor effects, during different tumor stages or in different interaction with other TME components [12]. A dynamic balance exists between the pro- and anti-tumor factors within the TME, which profoundly influences the prognosis of patients with cancer [13].

In order to identify the TME features of ESCA, RNA sequencing data and clinical information of ESCA samples were collected from The Cancer Genome Atlas (TCGA) databases. Additionally, we also identified the TME-associated differentially expressed genes (DEGs) which were related to prognosis. Based on them, the TME score was explored. Thus, our findings provide prognostic TME score and potential biomarkers which may assist oncologists in prognosis prediction.

## MATERIALS AND METHODS

### Collected esophageal cancer dataset

The RNA sequencing data (FPKM value) of gene expression in TCGA dataset were downloaded from the Genomic Data Commons (https://portal.gdc.cancer.gov/) using the R package TCGAbiolinks [14]. Then, FPKM values were transformed into transcripts per kilobase million (TPM) values. Data were analyzed with the R (version 3.6.1) and R Bioconductor packages.

### Collection of clinical data of esophageal cancer dataset

The clinical data and sample information for TCGA-ESCA and other TCGA cancer cohorts samples were obtained from the Genomic Data Commons (https://portal.gdc.cancer.gov/) using the R package TCGAbiolinks [14]. Overall survival (OS) information of all TCGA dataset was obtained from the supplementary data of published research. Somatic mutation data for ESCA patients were obtained from TCGA database (https://portal.gdc.cancer.gov/projects/TCGA-ESCA).

### Calculation of infiltrating cells in the TME

We used the Gene Set Variation Analysis (GSVA) [15] algorithm to analyze the gene signatures of the 28 TME cells based on the supplementary data from Jia, Q, et al. [16] .This algorithm allows for calculating the enrichment score of 28 human immune cell phenotypes, namely, activated B cell, immature B cell, mast cell, regulatory T cell, MDSC, effector memory CD8 T cell, central memory CD4 T cell, activated dendritic cell, macrophage, type 1 T helper cell, natural killer T cell, T follicular helper cell, natural killer cell, type 2 T helper cell, effector memory CD4 T cell, CD56 bright natural killer cell, gamma delta T cell, plasmacytoid dendritic cell, activated CD4 T cell, activated CD8 T cell, neutrophil, eosinophil, CD56dim natural killer cell, immature dendritic cell, central memory CD8 T cell, type 17 T helper cell, memory B cell and monocyte.

### Unsupervised clustering using TME infiltrating cells matrix

Tumors with qualitatively different TME cell infiltration patterns were grouped using hierarchical agglomerative clustering (based on canberra distance and Ward’s linkage (ward.D method)). Unsupervised clustering methods for dataset analysis were used to identify TME patterns and classify patients for further analysis. A consensus clustering algorithm was applied to determine the number of clusters to assess the stability of the discovered clusters. This procedure was performed using the ConsensusClusterPlus R package and was repeated 1,000 times to ensure the stability of classification [17].

### DEGs calculation and dimension reduction of different TME groups

To identify genes associated with TME cell enrichment score patterns, we grouped patients into TMEgroups based on immune-cell infiltration. DEGs among these groups were determined using the R package limma [18], which implements an empirical Bayesian approach to estimate gene-expression changes using moderated *t*-tests. DEGs among TME subgroups were determined by significance criteria (adjusted *P* value <0.05) as implemented in the R package limma. The adjusted *P* value for multiple testing was calculated using the Benjamini-Hochberg correction. An unsupervised clustering method (K-means) for analysis of DEGs was used to classify patients into several groups for further analysis. Then, the random forest classification algorithm was used to perform dimension reduction in order to reduce noise. Next, the clusterProfiler R package was adopted to annotate gene patterns [19]. After that, two TME signature gene sets (TME metagene 1 and TME metagene 2) were obtained to build the model of TME score.

### Calculation of TME score

GSVA was performed to calculate the signature score of different TME signature gene sets using the ssGSEA algorithm [15]. TMEscoreA and TMEscoreB represented the GSVA score calculated from different TME metagene 1 and TME metagene 2.


$$\begin{array}{c} \text{TMEscoreA}=\text{GSVA score of TME metagene 1}\\ \text{TMEscoreB}=\text{GSVA score of TME metagene 2} \end{array}$$

The prognosis of the patients with higher TMEscoreA was poor, and patients with lower TMEscoreB was poor. The stratification of prognosis for TMEscoreA and TMEscoreB is significant. After obtaining the prognostic value of each gene signature score, we applied a method to define the TMEscore of each patient:


TMEscore=TMEscoreB−TMEscoreA

### Functional enrichment analysis

The clusterProfiler R package was performed to demonstrate functional enrichment analysis on TME signature genes to. Gene Ontology (GO) and Kyoto Encyclopedia of Genes and Genomes (KEGG) terms were identified with a strict cutoff of *P* < 0.05 [20]. We also identified functional pathways that were up and down regulated among TMEscore high and low by running a gene set enrichment analysis (GSEA) of the adjusted expression data for all transcripts. Enrichment *P* values were based on 1,000 permutations and subsequently adjusted for multiple testing using the Benjamini-Hochberg procedure to control the FDR. A developing R package enrichplot (https://github.com/GuangchuangYu/enrichplot), implements several visualization methods to help interpreting enrichment results and is adopted to visualize GSEA result of TME gene groups. The hub genes were analyzed via STRING database (https://string-db.org/).

### Analysis of relevant biological processes using TME signatures

To explore the correlation between the TME signature and other relevant biological processes, we used gene sets curated by Mariathasan and colleagues, including (i) CD8 T-effector signature; (ii) antigen processing machinery; (iii) immune-checkpoint; (iv) epithelial-mesenchymal transition (EMT) markers previously reported (EMT1, EMT2, EMT3); (v) pan-fibroblast TGFb response signature (Pan-F-TBRS); (vi) Angiogenesis signature previously reported; (vii) Fanconi anemia; (viii) cell cycle genes (KEGG); (ix) DNA replication (KEGG); (x) nucleotide excision repair (KEGG); (xi) DNA damage repair (KEGG); (xii) Homologous recombination (KEGG); (xiii) mismatch repair (KEGG); (xiv) WNT targets; (xv) Cell cycle regulators (KEGG); (xvi) DNA damage repair (KEGG).

### Statistical analysis

Wilcoxon rank-sum test was used for the comparisons between two groups. The correlation coefficients were computed by Spearman and distance correlation analyses. Two-sided Fisher exact tests were used to analyze the contingency tables. The cutoff values of each dataset were evaluated based on the association between the OS of patient and TMEscore in each separate dataset using the survminer package. The R package MaxStat, which iteratively tests all possible cut points to find the one achieving the maximum rank statistic, was used to dichotomize TMEscore, and patients were then divided into low and high group. R package forestplot was used for the presentation of the results in the subgroup analysis of TMEscore in TCGA esophageal cancer dataset. To identify significant genes in the differential gene analysis, we applied the Benjamini-Hochberg method to convert the *P* values to FDRs. The Kaplan-Meier method was used to generate survival curves for the subgroups in each dataset, and the log-rank (Mantel-Cox) test was used to determine the statistical significance of differences. The hazard ratios for univariate analyses were calculated using a univariate Cox proportional hazards regression model. A multivariate Cox regression model was used to determine independent prognostic factors using the survminer package. All heat maps were generated by the function of heatmap (https://github.com/raivokolde/pheatmap). The *P* values were two-sided. *P* values of less than 0.05 were considered statistically significant.

### Data availability statement

The data used to support the findings of this study are all public and accessible at TCGA database.

## RESULTS

**Figure 1:**
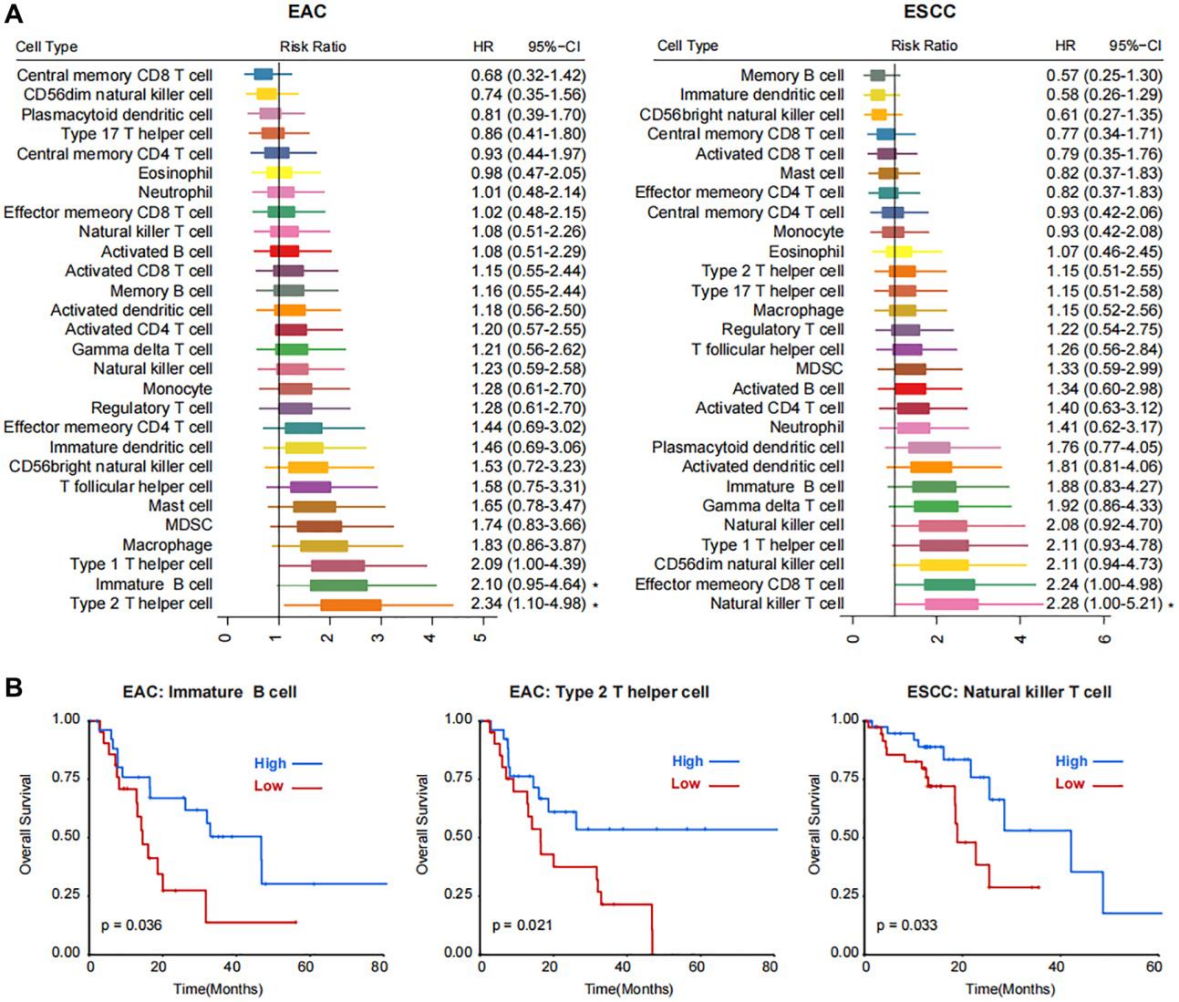
**Correlations between immune cell populations and the prognosis of two subtypes of esophageal cancer, EAC and ESCC.** (**A**) Forest plot showing the Risk Ratio and Hazard Ratio (HR) with the 95% CI of 28 kinds of TME cells in EAC and ESCC subtypes. Statistical significance is marked with asterisk at the right side. (**B**) Kaplan-Meier plots showing the survival curves of patients with high- and low- enrichment score of immature B cell and type 2 T helper cell in EAC and natural killer T cell in ESCC subtypes.

A total of 173 cases with available overall survival data were obtained from TCGA database via the association number TCGA-ESCA (Supplementary Figure 1). After removing the duplicate cases, 164 cases remained for further analysis. Based on the GSVA algorithm, 28 kinds of TME cells were calculated. The ESCA in TCGA database was divided into two subtypes, esophageal adenocarcinoma (EAC, *n* = 49) and esophageal squamous-cell carcinoma (ESCC, *n* = 78). The activated B cell was differentially expressed in the TME cell components between EAC and ESCC samples (Supplementary Figure 2). We next analyzed the clinical correlation of different TME cells in EAC and ESCC subtypes. In EAC, immature B cell and type 2 T helper cell played the main roles (Figure 1A). While in ESCC, natural killer T cells might be crucial (Figure 1A). Patients with higher enrichment score in immature B cell or type 2 T helper cell might have better clinical outcomes in EAC (*P* < 0.05, Figure 1B). In ESCC, the clinical outcome of patients with higher natural killer T cell was favorable (*P* < 0.05, Figure 1B). In the comprehensive analysis of all patients with ESCA, four kinds of TME cells, i.e. natural killer T cell, immature B cell, natural killer cell, and type 1 T helper cell, were significantly correlated with prognosis (Supplementary Figure 3).

**Figure 2:**
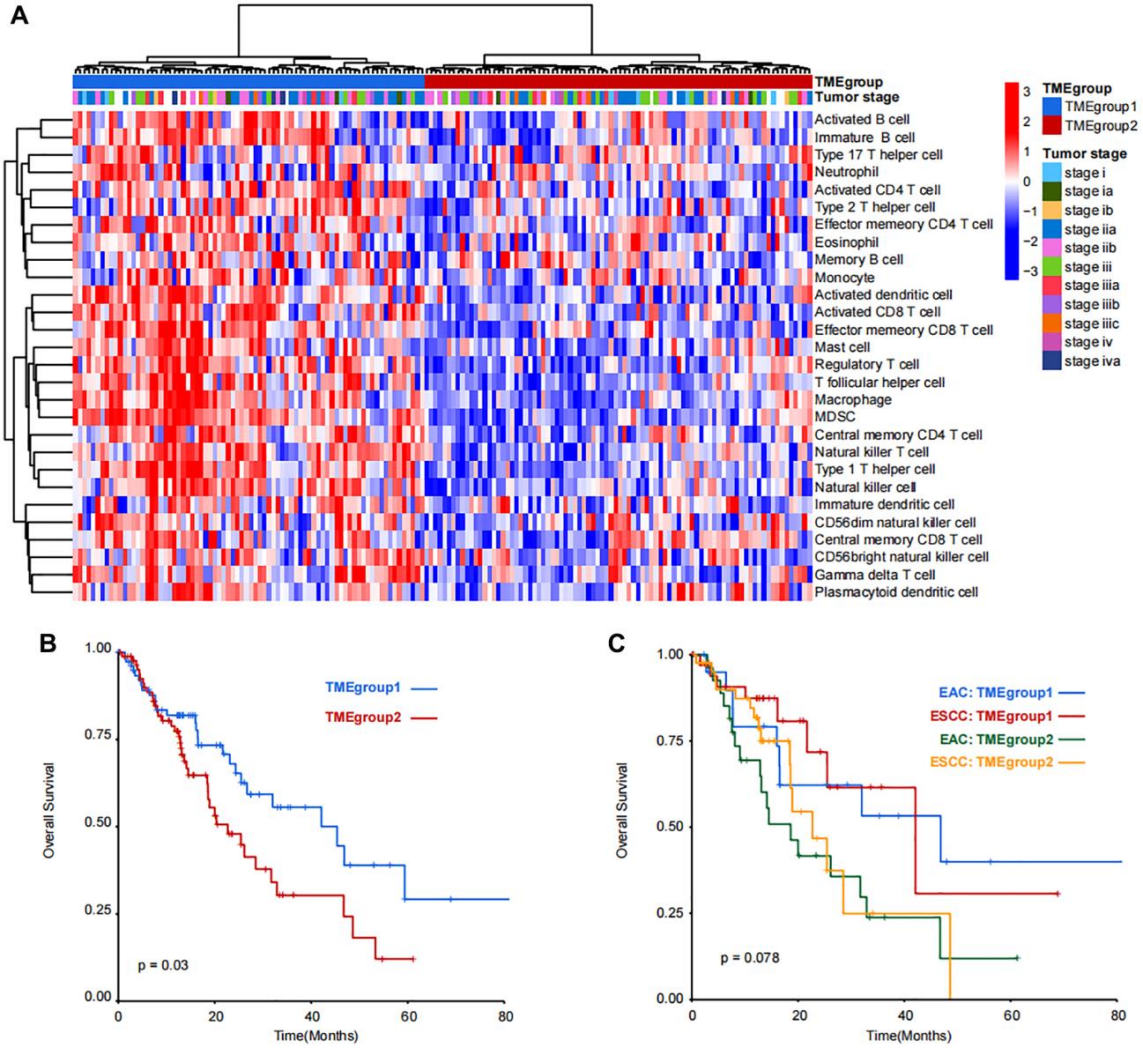
**Unsupervised clustering and prognostic analysis of immune cell populations in TCGA-ESCA dataset.** (**A**) Patients were divided into TMEgroup1 and TMEgroup2 by unsupervised clustering of 28 kinds of TME cells. TMEgroups and tumor stage of each patient are marked on the top of the heatmap. The color bar corresponds to the normalized enrichment score of TME cells. (**B**) Kaplan-Meier plot showing the overall survival of patients in TMEgroup1 (line in blue) and TMEgroup2 (line in red). (**C**) Kaplan-Meier plot showing the overall survival of patients in TMEgroup1 and TMEgroup2 between EAC and ESCC subtypes.

Due to little difference between TME cells in ESC and EACC, unsupervised clustering was applied in the 164 patients and two TME groups were identified (TMEgroup1 and TMEgroup2, Figure 2A). Subsequently, we tested four cluster number parameters in the analysis from k = 2 to k = 5 and the results of Consensus Cluster Plus revealed that 2 was the best cluster number (Supplementary Figure 4). By comparing the two TME groups, we found the higher enrichment score of 25 kinds of TME cells in TMEgroup1 (Figure 2A and Supplementary Figure 5). Additionally, the clinical outcome of TMEgroup1 was relatively favorable (*P* = 0.03, Figure 2B). The prognostic stratification based on TME cluster was not affected with esophageal cancer subtypes (*P* = 0.078, Figure 2C).

**Figure 3:**
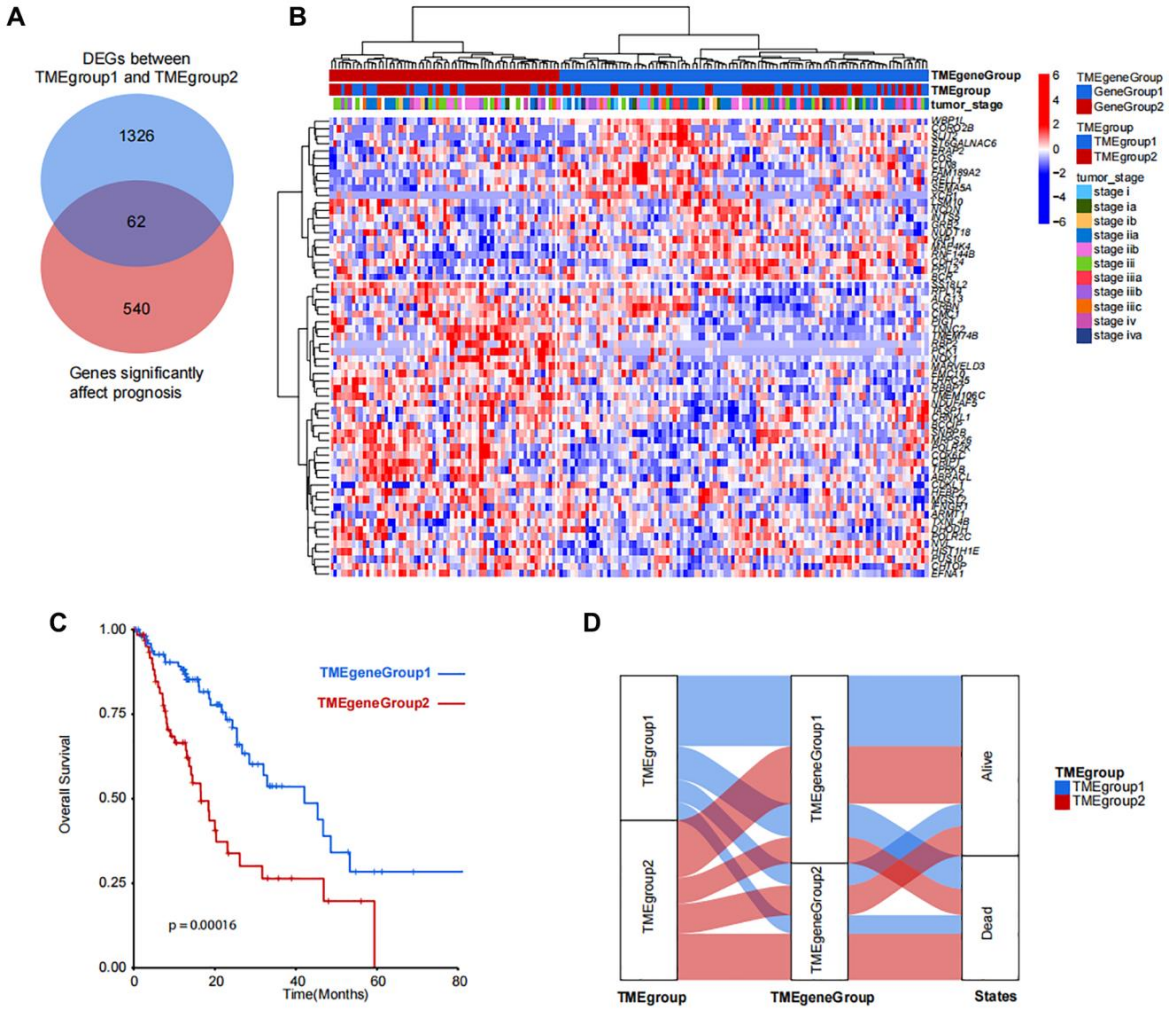
**Identification and clustering of TMEgroup and prognosis correlated genes.** (**A**) Venn diagram shows the number of genes significantly affect prognosis and DEGs between predefined TMEgroups. (**B**) Unsupervised clustering of 62 genes shows the 2 clusters of patients. TMEgeneGroups, TMEgroups and tumor stage of each patient are marked on the top of the heatmap. The color bar corresponds to the normalized expression value of signature genes. (**C**) Kaplan-Meier plot showing the overall survival of TMEgeneGroup1 (line in blue) and TMEgeneGroup2 (line in red). (**D**) Sankey diagram showing the proportional relationship in TMEgroup substyles, TMEgenegroup substyles and the patient survival states.

In order to analyze the difference between TMEgroup1 and TMEgroup2, we identified the DEGs between the two TME groups. Then, the correlation between the DEGs and clinical prognosis were explored and a total of 62 genes were finally identified (Figure 3A). Based on the expressions of these 62 genes, the patients could be divided into two groups (TMEgeneGroup1 and TMEgeneGroup2) (Figure 3B). The gene patterns between two TME gene group could also be divided into two different parts, *SS18L2*, *RPL14*, *ALG13*, *CRBN*, *CMC1*, *PIGT*, *TNNC2*, *TMEM74B*, *RBP2*, *PCK1*, *NOX1*, *MARVELD3*, *EMC10*, *LRRC45*, *RBBP7*, *TMEM106C*, *NDUFAF5*, *TASP1*, *CRNKL1*, *BCCIP,* *SNRPB*, *MRPS26*, *POLR2K*, *COX6C*, *CRIPT*, *TPRKB*, *ABRACL*, *CDKL1*, *HEBP2*, *MGST2*, *FNGR1*, *ARMT1*, *TXNL4B*, *DHODH*, *POLR2C*, *NVL*, *HIST1H1E*, *PUS10*, *CHTOP*, *EFNA1* belonged to the gene signature of TMEgeneGroup1 (TME metagene 1), and were highly expressed in TMEgeneGroup1, while *WBP1L*, *CORO2B*, *SLIT2*, *ST6GALNAC6*, *ERAP2*, *FOS*, *CLN8*, *FAM189A2*, *RELL1*, *SEMA5A*, *XCR1*, *LSM10*, *NCDN*, *INTS5*, *GRB2*, *NUDT18*, *YAP1*, *MAP4K4*, *RNF144B*, *CDH24*, *PPIL2*, *BCR* belonged to the gene signature of TMEgeneGroup2 (TME metagene 2), and were highly expressed in TMEgeneGroup2 (Figure 3B). The clinical outcomes were also found between the two groups and TMEgeneGroup2 had a poor prognosis (*P* = 0.00016, Figure 3C). Most patients in TMEgroup1 were clustered into TMEgeneGroup1, and the prognostic difference between TMEgeneGroup were more obvious in that between TMEgroups (Figure 3D). The TMEgeneGroups were significantly correlated with TMEgroup (*p*-value = 0.01, Pearson’s Chi-squared Test). The individual information for both TMEgroup, TMEgeneGroup and all clinical traits was presented in the Supplementary Table 1. The prognostic stratification revealed that in TMEgeneGroup2, the prognosis of patients with either EAC or ESCC was poor (Supplementary Figure 6).

**Figure 4:**
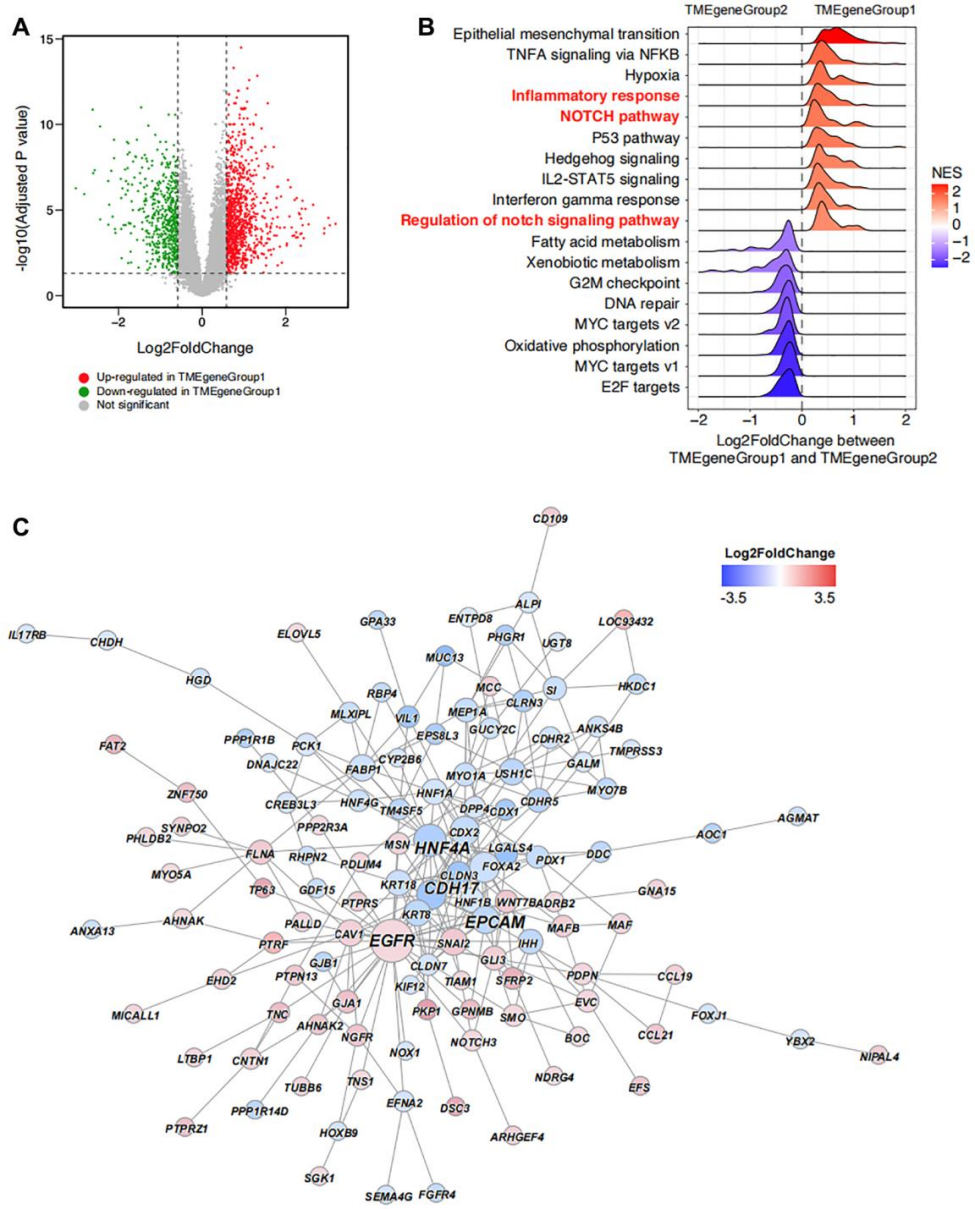
**Unsupervised clustering enrichment analysis of DEGs.** (**A**) DEGs between TMEgeneGroup1 and TMEgeneGroup2. The x axis corresponds to log2 transformed fold change value, and the y axis corresponds to –log10 transformed adjusted *P* value. Up- and down- regulated genes in TMEgeneGroup1 are shown as red and blue dots. (**B**) GSEA analysis shows the top enriched pathways between TMEgeneGroup1 and TMEgeneGroup2. Pathways were ordered by NES and all pathways had P values less than 0.05. (**C**) PPI network of the DEGs between TMEgeneGroup1 and TMEgeneGroup2. Up- and down- regulated genes in TMEgeneGroup1 are colored in red and blue. Size of nodes and gene labels correspond to the hub score of genes.

The DEGs between TMEgeneGroup1 and TMEgeneGroup2 was also calculated with down-regulated 546 genes in TMEgeneGroup and up-regulated 1028 genes in TMEgeneGroup2 (Figure 4A). The results form Gene Set Enrichment Analysis (GSEA) showed that epithelial mesenchymal transition, TNFA signaling via NFKB, inflammatory response, NOTCH signaling pathway were significantly up-regulated in TMEgeneGroup1, indicating that patients in TMEgeneGroup1 were active in immune response and the function of immune system were more efficient (Figure 4B). And to TMEgeneGroup2, G2M checkpoint, MYC targets v2 and oxidative phosphorylation were significantly up-regulated, indicating that cell cycle and tumor progression were activated in this group (Figure 4B). Moreover, *EGFR* was significantly up-regulated in TMEgeneGroup1 by the hub gene analysis, and it might be a therapeutic target in ESCA (Figure 4C). Other three hub genes, *HNF4A*, CDH17 and *EPCAM* were up-regulated in TMEgeneGroup2, and might serve as the biomarkers of TMEgeneGroup2 (Figure 4C).

Based on the 62 genes calculated from TMEgeneGroup, we built a model to increase the prediction efficiency. In addition, we also calculated TMEscoreA using TME metagene 1, and TMEscoreB using TME metagene 2 according to ssGSEA algorithm. Through the comprehensive analysis, we found that the clinical (*P* < 0.0001, Supplementary Figure 7A) and the clinical outcome of patients with higher TMEscoreB was favorable (*P* < 0.0001, Supplementary Figure 7B).

**Figure 5:**
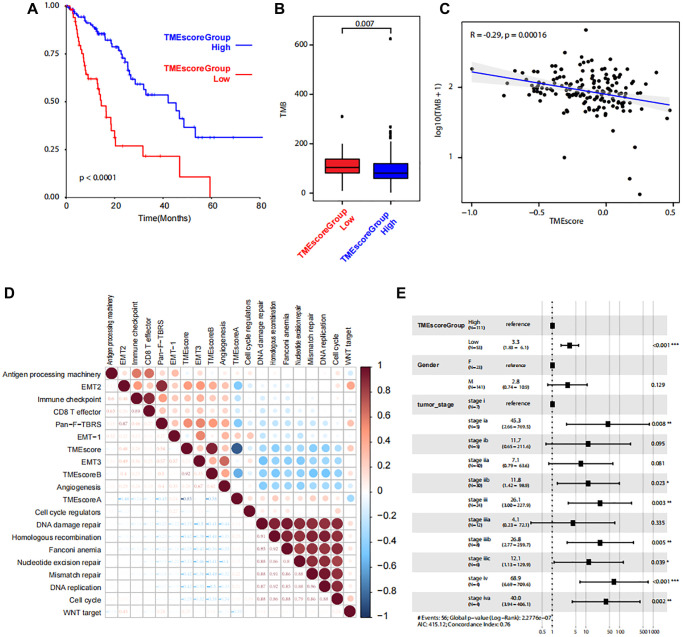
**Correlations between TMEscore and patient prognosis.** (**A**) Kaplan-Meier plot shows significant difference of the overall survival between the two groups with high and low level of TME scores. (**B**) Box plot shows TMB between the two groups with high and low level of TME scores. (**C**) Line regression shows the correlation between TMEscore and TMB. R value and *P* values are also labelled. (**D**) The correlation between cancer related pathways and TMEscore. The size and color correspond to the correlation values. (**E**) The multivariate Cox regression model shows the correlations between TMEscore and clinical phenotypes.

Based on the TMEscoreA and TMEscoreB, we calculated the final TMEscore and we identified that the clinical outcome of patients with higher TMEscore was favorable (*P* < 0.0001, Figure 5A). Also, the tumor mutation burden (TMB) in low TME score group was higher than that in high TME score group (Figure 5B). The correlation analysis revealed a significant correlation between TMEscore and TMB (Figure 5C). After filtration of gene mutation profile between low TMEscore and high TMEscore, we found that the mutation frequency of *SYNE1* was higher in low TME score (Supplementary Figure 8). Gene expression level of 17 pathways were also analyzed to identify the correlation between pathways and TMEscore, and the results revealed the positive correlation with EMT2, immune checkpoint, and Pan-F-TBRS, and negative correlation with DNA damage repair, homologous recombination, Fanconi anemia, nucleotide excision repair, mismatch repair, DNA replication and cell cycle (Figure 5D). Multifactor analysis showed TMEscore was an independent prognostic factor for the prognosis of patients with ESCA (Figure 5E).

**Figure 6:**
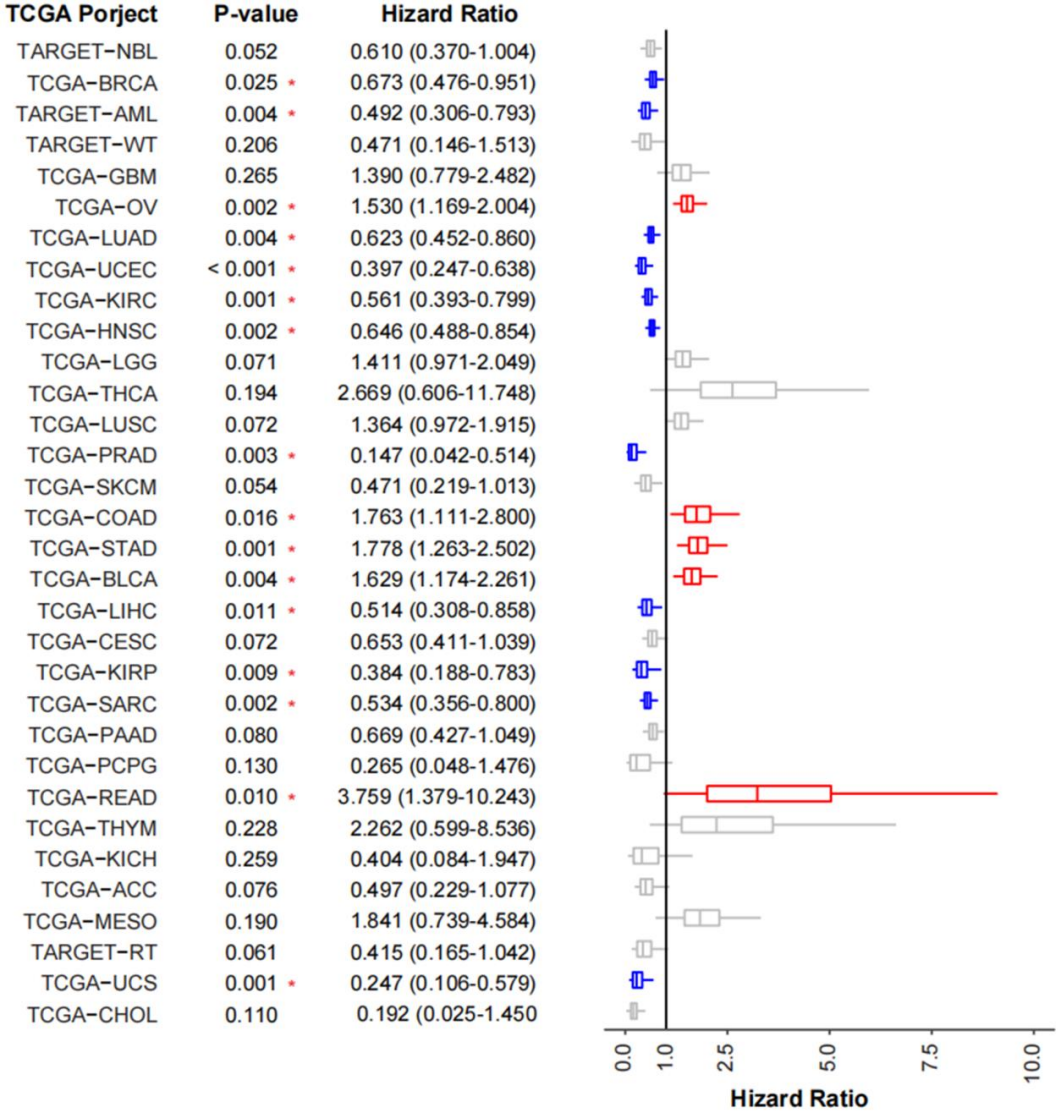
**Validation of TMEscore on TCGA cancer cohorts.** Forest plot showing the prognosis of TMEscore in 32 tumor cohorts of TCGA. The HR of tumor types marked in blue represent the prognosis of high-TMEscore group is significantly good than that in the low TMEscore group, while those marked in red are opposite.

To validate the prognostic impact of TMEscore model in cancer cohorts, we use 32 TCGA cancer cohorts (TCGA-ESCA was not included) and calculated TMEscoreA and TMEscoreB of each patient. Generally, 11 (34.3%) cohorts (TCGA-PRAD, TCGA-UCS, TCGA-KIRP, TCGA-UCEC, TARGET-AML, TCGA-LIHC, TCGA-SARC, TCGA-KIRC, TCGA-LUAD, TCGA-HNSC, TCGA-BRCA) revealed the TMEscore as a good prognostic indicator, whereas 5 (15.6%) cohorts (TCGA-OV, TCGA-BLCA, TCGA-COAD, TCGA-STAD, TCGA-READ) revealed as a poor prognostic indicator (Figure 6). By validating in other TCGA cancer cohorts, we found most cohorts (21/32 of TCGA cohorts had HR less than 1 and 11/32 were significant) had a similar prognostic trend using TMEscore, revealing TMEscore as a helpful indicator for tumorigenic microenvironment and prognosis.

## DISCUSSION

ESCA has a high mortality rate and poor prognosis, with an estimated 18,440 new cases and 16,170 deaths projected in 2020 in the United States [2]. TME plays an important role in the tumorigenesis, progression and therapy resistance of ESCA [21, 22]. Thus, there is a pressing need to explore the TME features which may assist oncologists in prognosis prediction. In this study, we identified the key immune cells (natural killer T cell, immature B cell, natural killer cell, and type 1 T helper cell) associated with the prognosis and found that the TME features was significantly related to the OS of patients with ESCA. Based on the key TME genes, we explored a TMEscore which was demonstrated to be a reliable index for predicting the clinical outcome for ESCA patients.

Generally, TME cells include inflammatory cells, such as T cells, B cells, dendritic cells, natural killer cells, neutrophils, macrophages, etc. The tumorigenesis of ESCA are etiologically related to the exposure of the refluxed gastric and bile acids, which trigger chronic inflammation and the occurrence of Barrett’s esophagus [23]. Other risk factors for ESCA, such as smoking and alcohol, also induce the toxic effects to increase the inflammation in the esophageal epithelium and subsequent play carcinogenic roles [24]. Thus, the ability of inflammatory responses may take part in the regulation of ESCA initiation and development. In addition, tumor cells can also recruit different immune cell populations or express immune checkpoints to suppress the anti-tumor immune response [21, 22]. Targeting immune checkpoints or supplement of the antitumor immune cells have been candidate options for anti-cancer therapy [8]. In this study, we found that natural killer T cell, immature B cell, natural killer cell and type 1 T helper cell were favorable for the prognosis of patients with ESCA.

Immune response related to the helper T cells is often impaired in ESCC patients and further damage the anti-tumor immunity [25]. Therapeutically, the double radiofrequency hyperthermia could regulate the conversion from Th2 to Th1 cells and work in the treatment of ESCA [26]. Impairment of natural killer cell activity also promotes tumor immunoevasion. T cell immunoglobulin domain and mucin domain-3 (Tim-3) was closely related to tumor invasion and distant metastasis in ESCC [27]. The mechanism was supposed to be associated with NK cell dysfunction in tumor microenvironment [28]. Besides, invariant natural killer T (iNKT) based immunotherapies showed promise for cancer patients and may benefit ESCA patients [29].

In view of the important roles of TMB in the tumorigenesis, progression and treatment, we explored a TMEscore based on the key TME genes and verified it as a reliable index for the clinical outcome and TMB. Evaluation of prognostic factors is important for oncologists in clinical decision-marking; thus many previous studies have focused on the identification of predictors for patients with ESCA. Clinical information (such as age, sex, tumor stage and grade), blood examination (such as C-reactive protein, albumin ratio), tumor characteristics (such as metabolic tumor volume and total lesion glycolysis) and treatment methods (such as chemoradiotherapy, chemotherapy and surgery) have been regarded as prognostic factors and proved to be associated with the prognosis of patients with ESCA [30–33]. However, these prognostic factors were most clinical and did not include the molecular biomarkers. With the well-application of TCGA dataset, regulatory network of microRNA, lncRNA, mRNA and has been identified and prognosis-associated biomarkers have also been evaluated for patients with ESCA [34–36]. Unfortunately, the tumor microenvironment scores have not been explored comprehensively. Thus, this study is a good supplement to the existing research about the prognosis evaluation of patients with ESCA.

In the TMEgeneGroup1 with favorable prognosis, inflammatory response and NOTCH signaling pathway were enriched. Notch is a key modulator in regulating T-cell development, maintenance, activation and takes part in T cell-mediated immune responses [37, 38]. In addition, *EGFR* was significantly up-regulated in TMEgeneGroup1 by the hub gene analysis. As a therapeutic target, *EGFR* amplifications can be found in ESCA and the application of *EGFR* inhibitors may improve the prognosis of these ESCA patients [39, 40]. Thus, the favorable prognosis of patients in the TMEgeneGroup1 may be due to the good immune response and the application of *EGFR* inhibitors.

This study inevitably has some limitations that should be declared. Firstly, due to limited published data and fewer cases of ESCA, the amount of data analyzed in this study is limited, which may lead to potential errors or deviations. We used TCGA cohorts to validate the prognosis impact of TMEscore. And in future work, more esophageal cancer patients would be enrolled and studied to deeply explore the tumor microenvironment and help to improve the prognosis. Secondly, as a bioinformatics analysis, the roles of identified immune cells is not verified by cell and molecular experiments or clinical tissue microarray. Thirdly, all data series downloaded in this study are from America, and we are not quite sure about its applicability in European and Asian.

## CONCLUSIONS

Our data provided key immune cells (natural killer T cell, immature B cell, natural killer cell, and type 1 T helper cell) associated with the prognosis of patients with ESCA and constructed a novel prognostic model, termed TMEscore, as a reliable index for predicting the clinical outcomes of ESCA patients. And by validation of other TCGA cancer cohorts, it revealed TMEscore could be applied to predict the prognosis of other tumors.

## Supplementary Materials

Supplementary Figures

Supplementary Table 1
