# Supplementary material for: Tumor microenvironment characterization in esophageal cancer identifies prognostic relevant immune cell subtypes and gene signatures
Source: Aging (Albany NY). 2021 Dec 26;13(24):26118–36. doi: 10.18632/aging.203800 (PMC8751614; doi:10.18632/aging.203800)
Supplement: Supplementary Figures [file aging-13-203800-s001.pdf]

SUPPLEMENTARY FIGURES

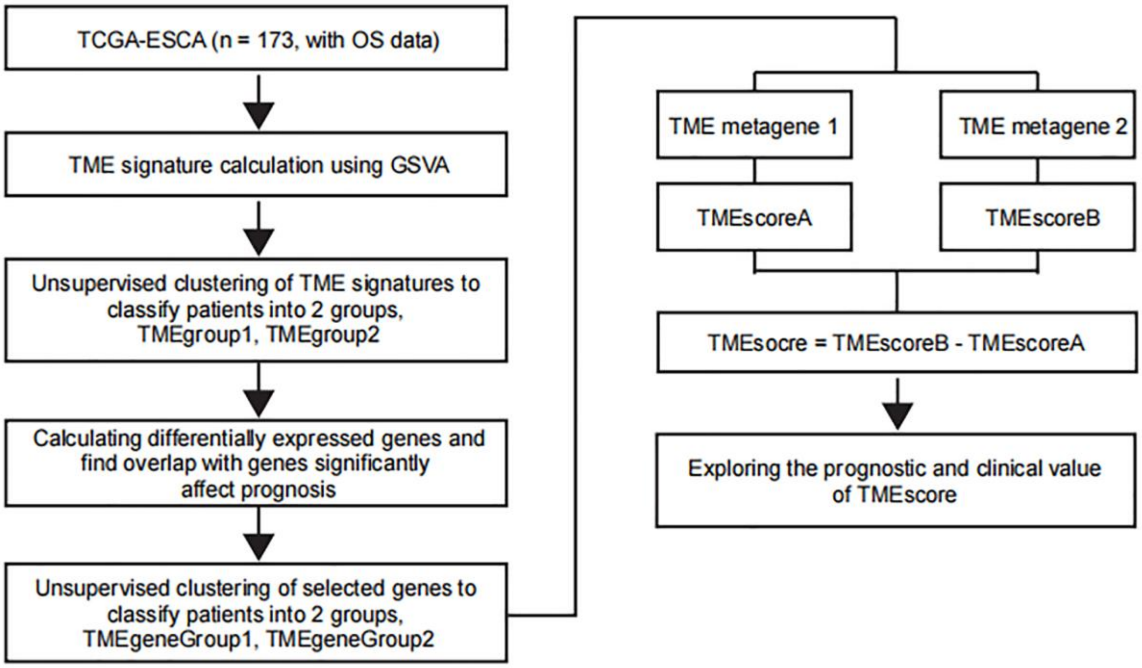

Supplementary Figure 1. The overall workflow of the study. Important analysis steps and datasets are highlighted in squares and the arrows indicates the direction of the pipeline.

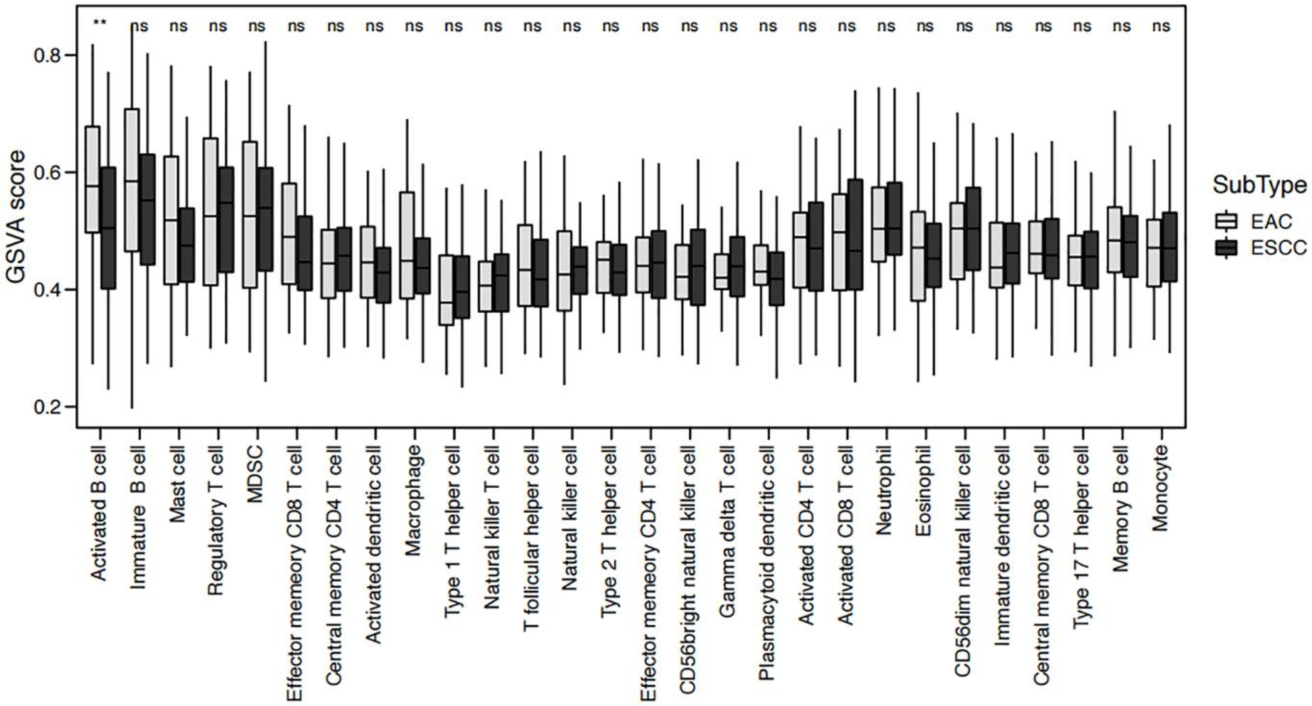

Supplementary Figure 2. Difference of TME cells between EAC and ESCC samples. Box plot shows the GSVA score of 28 kinds of TME cells. EAC and ESCC patients are colored in light grey and black. The statistical significance are also labelled. ns for not significant. ** for $p < 0.01$.

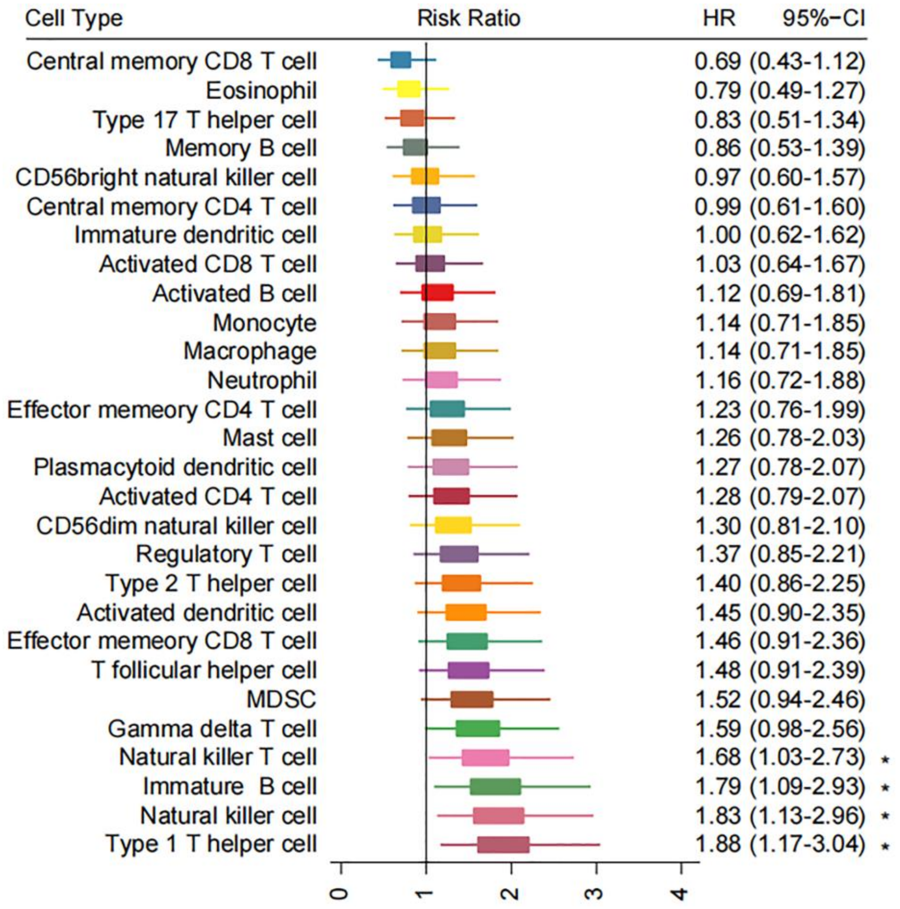

Supplementary Figure 3. Correlations between TME cells and clinical outcomes. Forest plot shows the Risk Ratio and Hazard Ratio with the 95% CI of 28 kinds of TME cells in all patients with ESCA. Statistical significance are marked with asterisk.

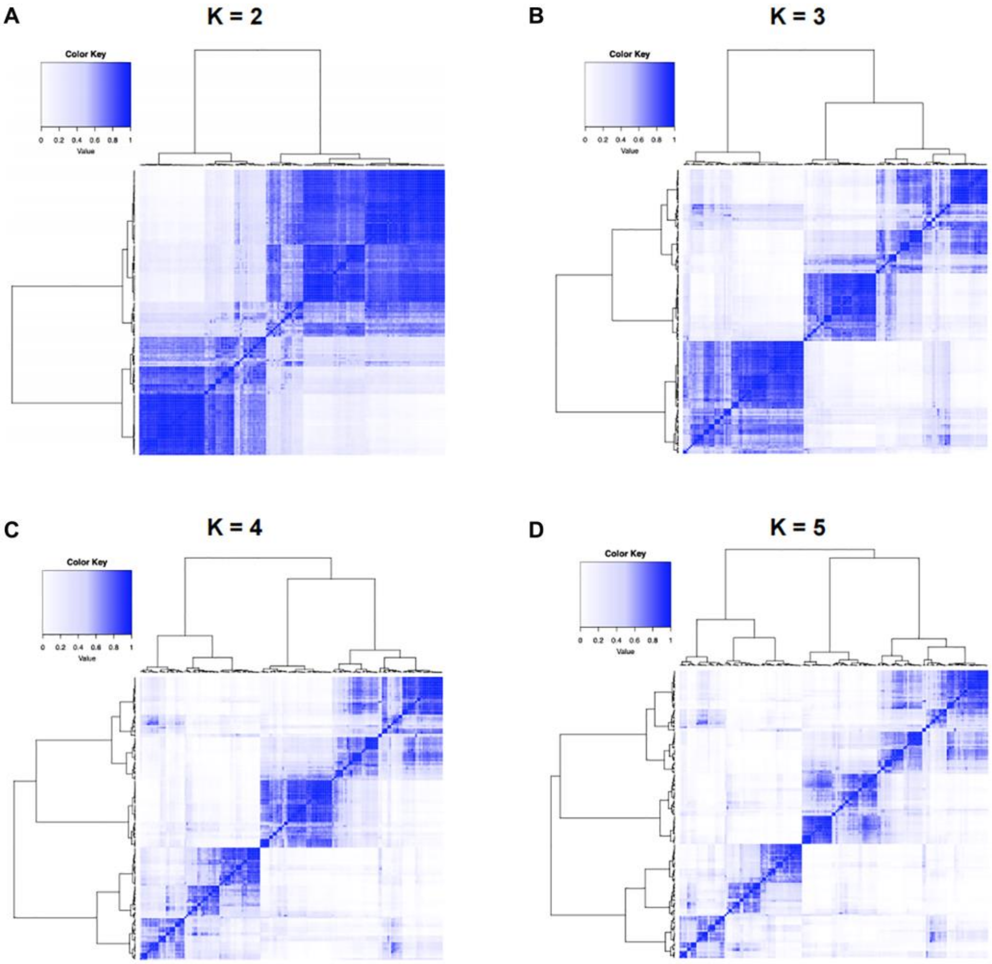

Supplementary Figure 4. Identification of consensus clusters by TME cells. Consensus clustering matrix for K = 2 (**A**), K = 3 (**B**), K = 4 (**C**), and K = 5 (**D**) are shown.

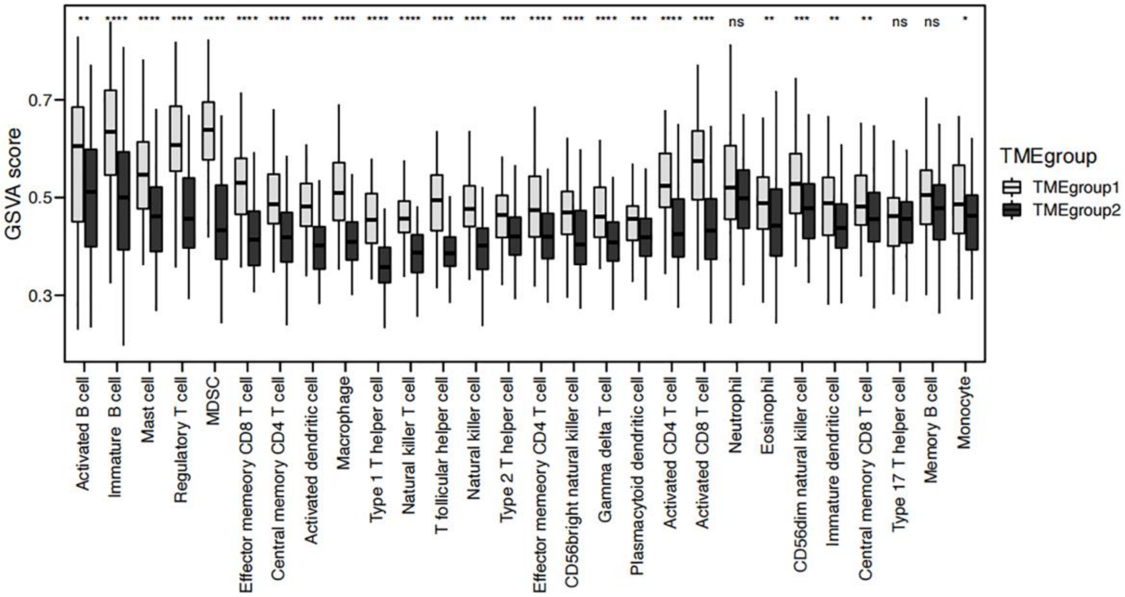

Supplementary Figure 5. Difference of TME cells between TMEgroup1 and TMEgroup1. Box plot shows the GSVA score of 28 kinds of TME cells. TMEgroup1 and ESCC TMEgroup1 are colored in light grey and black. The statistical significance are also labelled. ns for not significant, * for $p < 0.05$, ** for $p < 0.01$, *** for $p < 0.001$ and **** for $p < 0.0001$.

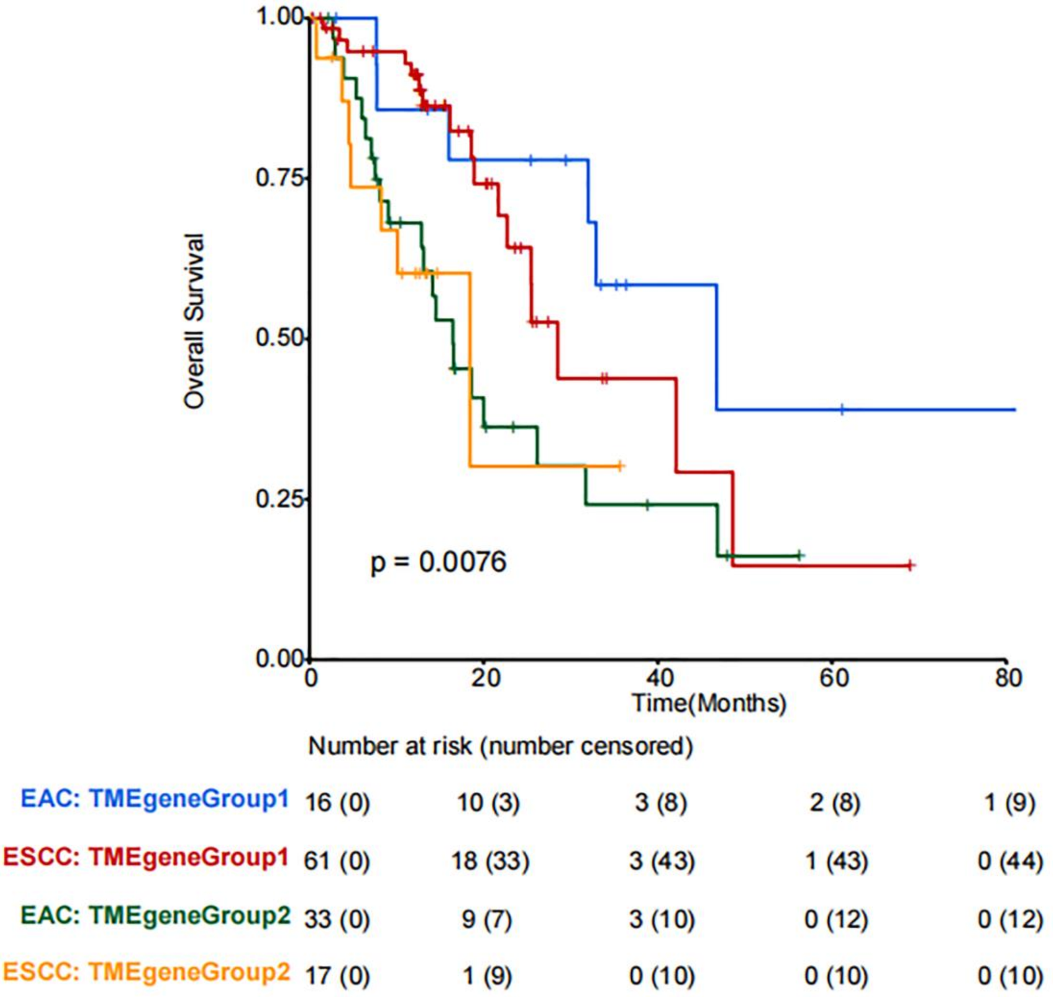

Supplementary Figure 6. Survival analysis of patients between different TMEgeneGroups and ESCA subtypes. Kaplan-Meier plot shows the overall survival among the patients within four groups of different TMEgeneGroups and ESCA subtypes. Survival curves are marked in different colors and the number of cases at risk and censored at different time points are also labelled.

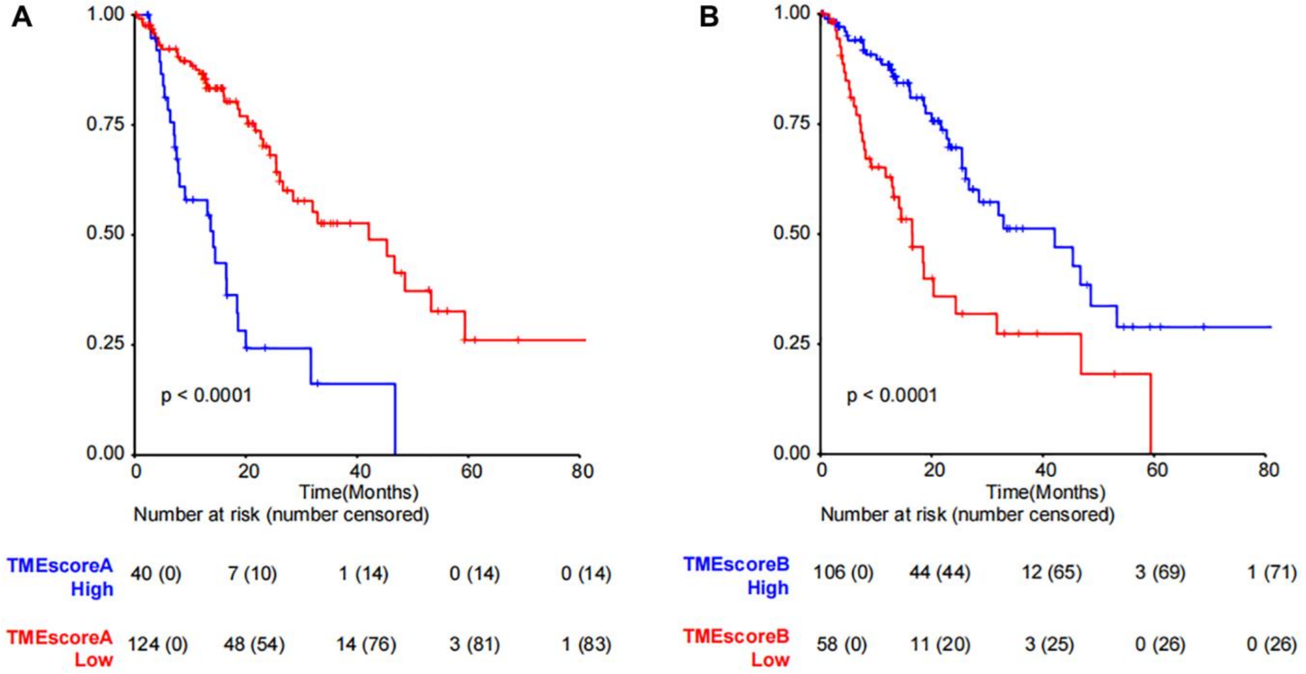

Supplementary Figure 7. Survival analysis of patients between different levels of TMEscore. Kaplan-Meier plot shows the overall survival among the patients within high and low levels of TMEscoreA (**A**) and TMEscoreB (**B**). Survival curves with high and low levels of TMEscore are colored in blue and red. The number of cases at risk and censored at different time points are also labelled.

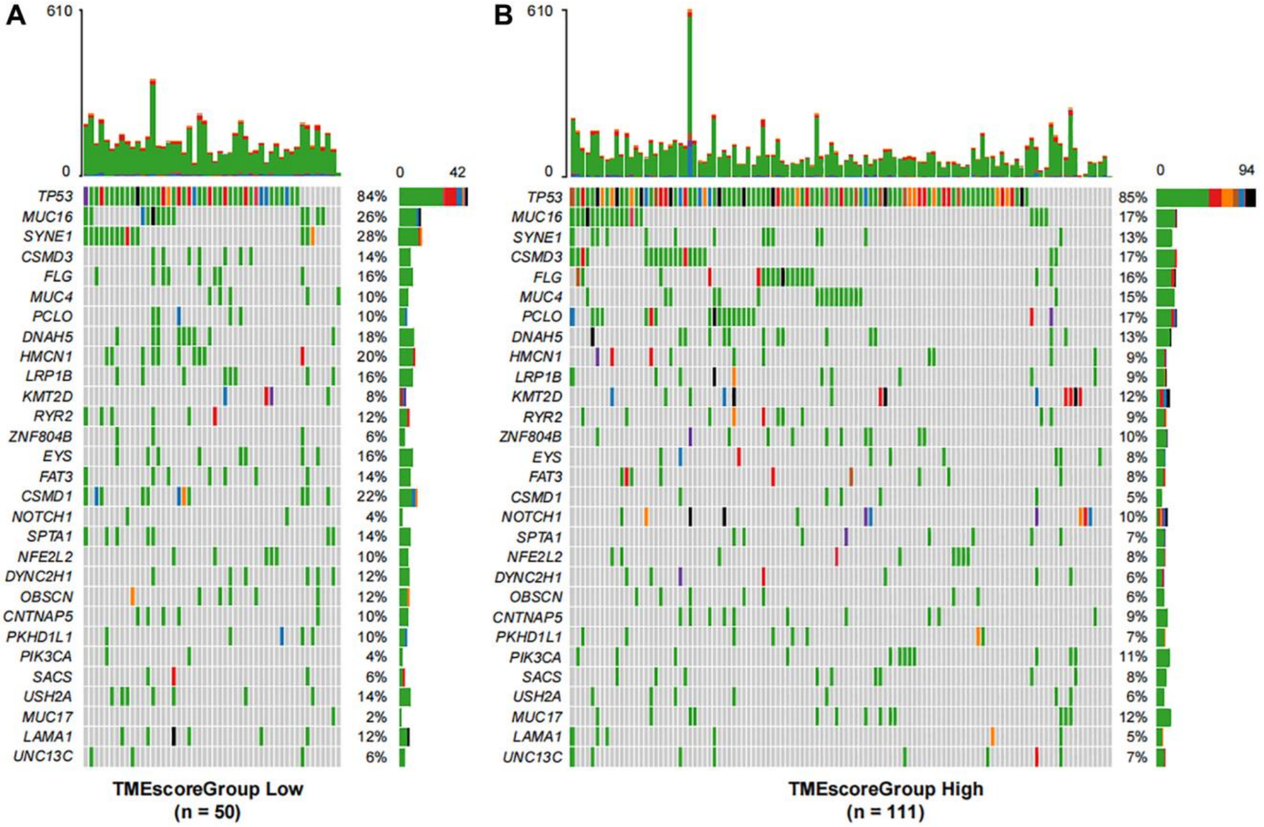

Supplementary Figure 8. Gene mutation profiles of patients with high and low TMEscore. CoMut plot shows the mutation profiles of top ranked mutated genes in TMEscoreGroup Low (**A**) and TMEscoreGroup High (**B**). The column corresponds to a patient, and the row corresponds to a gene. The bar plot on the top shows the number of total mutations within each patient, and the bar plot on the right panel shows the number of different mutations with a gene among all patients. The mutation frequencies of genes are also labelled.
