# Supplementary figures and images for: Tumor microenvironment characterization in esophageal cancer identifies prognostic relevant immune cell subtypes and gene signatures
Source: Aging (Albany NY). 2021 Dec 26;13(24):26118–36. doi: 10.18632/aging.203800 (PMC8751614; doi:10.18632/aging.203800)

Supplementary Table 1. Information of each individual in TCGA-ESCA dataset.


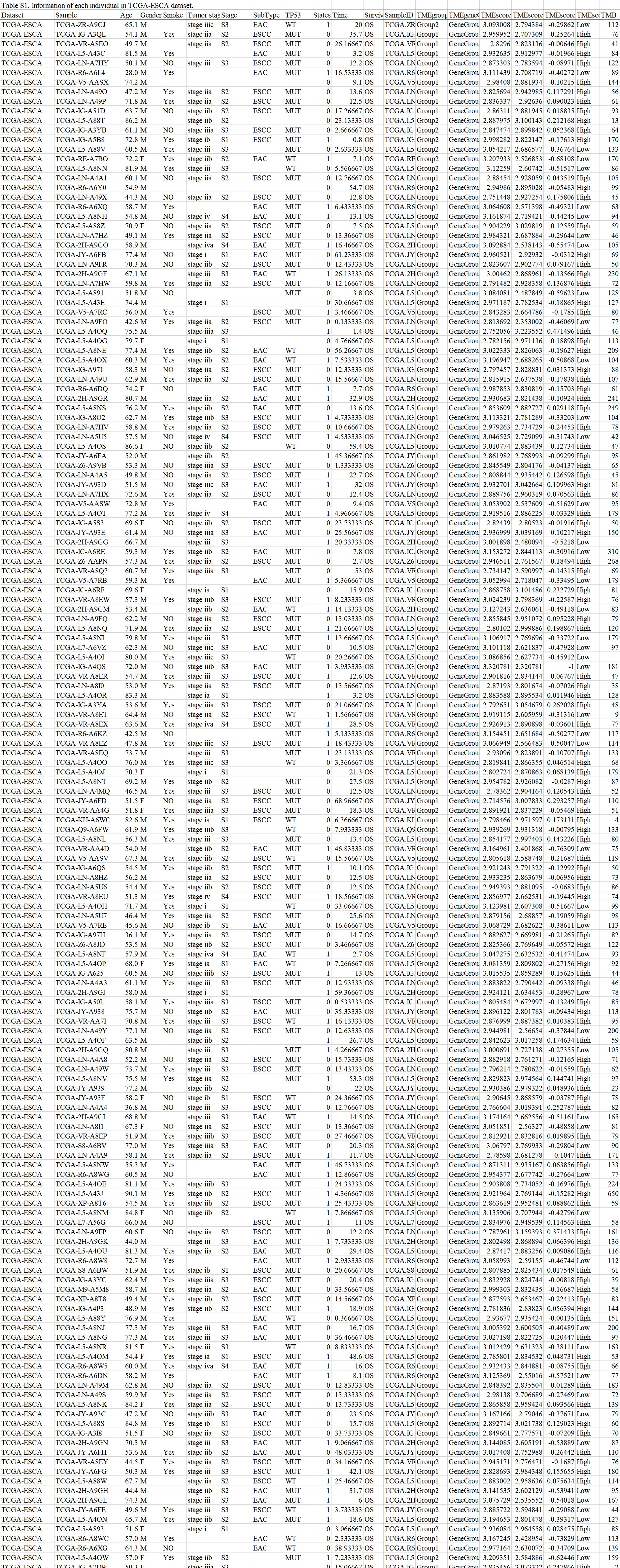

Supplement: Supplementary Table 1 [file aging-13-203800-s002.docx]
